# Kinematic predictors of single-leg squat performance: a comparison of experienced physiotherapists and student physiotherapists

**DOI:** 10.1186/1471-2474-13-207

**Published:** 2012-10-25

**Authors:** Benjamin K Weeks, Christopher P Carty, Sean A Horan

**Affiliations:** 1 Centre for Musculoskeletal Research, Griffith Health Institute, Griffith University, Gold Coast, Queensland, Australia; 2 School of Rehabilitation Sciences, Griffith University, Gold Coast, QLD, 4222, Australia

**Keywords:** Kinematics, Lower limb, Physiotherapy, Reliability, Single leg squat

## Abstract

**Background:**

The single-leg squat (SLS) is a common test used by clinicians for the musculoskeletal assessment of the lower limb. The aim of the current study was to reveal the kinematic parameters used by experienced and inexperienced clinicians to determine SLS performance and establish reliability of such assessment.

**Methods:**

Twenty-two healthy, young adults (23.8 ± 3.1 years) performed three SLSs on each leg whilst being videoed. Three-dimensional data for the hip and knee was recorded using a 10-camera optical motion analysis system (Vicon, Oxford, UK). SLS performance was rated from video data using a 10-point ordinal scale by experienced musculoskeletal physiotherapists and student physiotherapists. All ratings were undertaken a second time at least two weeks after the first by the same raters. Stepwise multiple regression analysis was performed to determine kinematic predictors of SLS performance scores and inter- and intra-rater reliability were determined using a two-way mixed model to generate intra-class correlation coefficients (ICC_3,1_) of consistency.

**Results:**

One SLS per leg for each participant was used for analysis, providing 44 SLSs in total. Eight experienced physiotherapists and eight physiotherapy students agreed to rate each SLS. Variance in physiotherapist scores was predicted by peak knee flexion, knee medio-lateral displacement, and peak hip adduction (R^2^ = 0.64, p = 0.01), while variance in student scores was predicted only by peak knee flexion, and knee medio-lateral displacement (R^2^ = 0.57, p = 0.01). Inter-rater reliability was good for physiotherapists (ICC_3,1_ = 0.71) and students (ICC_3,1_ = 0.60), whilst intra-rater reliability was excellent for physiotherapists (ICC_3,1_ = 0.81) and good for students (ICC_3,1_ = 0.71).

**Conclusion:**

Physiotherapists and students are both capable of reliable assessment of SLS performance. Physiotherapist assessments, however, bear stronger relationships to lower limb kinematics and are more sensitive to hip joint motion than student assessments.

## Background

The single-leg squat (SLS) is a functional clinical test used by physiotherapists in the musculoskeletal assessment of the lower limb. Despite ubiquity of use, little is known of the validity and reliability of the test. Specifically, the relationship that physiotherapy assessment bears with kinematic parameters is unclear. Furthermore, the influence of clinician experience on the validity and reliability of SLS assessment is yet to be established. Thus, a comprehensive investigation of SLS kinematics in light of experienced and inexperienced physiotherapist assessment is warranted to justify the utility of the test.

There is some evidence that kinematic patterns, particularly at the knee, are characteristic of musculoskeletal conditions or related to lower limb injury risk [1-3]*.* An increase in knee valgus angle during functional tasks, for example, is recognised as an important risk factor for non-contact anterior cruciate ligament injury [1,4]. Similarly, increased knee valgus angle during weight-bearing tasks is capable of separating individuals with and without patellofemoral pain syndrome [2]. Indeed, dynamic knee valgus observed in the frontal plane during common athletic screening tasks is a reliable method of identifying those at high risk of knee injury [5]. Others have identified relationships between excessive knee frontal plane motion and hip muscle weakness [6]. For clinicians, the application of these findings to practice relies on an ability to recognise abnormal movement patterns through visual observation during the clinical exam.

The SLS test is touted to be a simple method of identifying clinically abnormal movement patterns to assist clinicians with screening and diagnosis. As such, a modicum of descriptive reports on the kinematics of the SLS have emerged [6-17], however many of these reports are focused on laboratory measures and their results may not directly translate to the clinical assessment of SLS performance [6,8,11,12,15,16]. Several research groups, however, have considered the nexus of laboratory findings and clinical implications by assessing the relationship between lower limb SLS kinematics and the clinical assessment of SLS performance. Ageberg and colleagues [7] found that the degree of knee medio-lateral motion could be reliably determined by musculoskeletal physiotherapists. Further, Crossley and colleagues [10] found that physiotherapists were able to use the SLS test as a reliable tool for identifying hip muscle dysfunction in asymptomatic participants. DiMattia and colleagues [17] however, were ambivalent of the associations between SLS assessment and its relationship with hip joint kinematics and abductor strength [17]. Rating method was investigated by Chmielewski and co-workers [9], who found that the assessment of SLS movement quality using an ‘overall approach’ rather than a ‘specific/segmental approach’ resulted in greater percent agreement between physiotherapists. The impact of experience on clinician reliability and validity of SLS assessment, however, remains unknown.

Though a small body of work is available lending support to the SLS test and its sensitivity to injury identification, most reports are limited to two-dimensional descriptions of kinematics or confined to a single joint. Furthermore, investigations to date have not considered the association between joint kinematics and clinician assessment or the influence of clinical experience. Therefore, the purpose of the present study was to determine kinematic predictors of perceived SLS quality for experienced and student physiotherapists and assess inter- and intra- rater reliability of SLS performance rating for experienced and student physiotherapists. We hypothesised that (i) physiotherapist and student ratings of SLS performance would be predicted by hip and knee joint kinematic variables and (ii) physiotherapist ratings of SLS performance would be more reliable than physiotherapy student ratings.

## Methods

### Study design

A cross-sectional study design was used to analyse SLS performance in healthy, young adults. Three-dimensional motion analysis was employed to determine lower limb joint kinematics and two-dimensional video capture was used to record SLSs for later rating. Physiotherapists and physiotherapy students were additionally recruited to rate the quality of SLS performance for comparison with kinematic data and to determine rater reliability. Written informed consent was obtained from each volunteer before data collection, and all experimental procedures were approved by the Griffith University Human Research Ethics Committee.

### Participants

We recruited healthy, young men and women to perform a series of SLSs. Participants were included if they were generally healthy, ambulatory, and between the ages of 18 and 35 years. Participants were excluded if they had any of the following: recent surgery or injury to the lower limb; previous lower limb orthopaedic surgery; balance or co-ordination impairment; or were taking medications known to impair movement or balance. We further recruited experienced musculoskeletal physiotherapists and physiotherapy students to rate the SLS performances. For the purpose of this study physiotherapists were deemed experienced if they had completed a post-graduate musculoskeletal physiotherapy qualification and had at least five years post-registration clinical experience. Students were included if they were currently enrolled in the second-last year of the physiotherapy program at Griffith University.

### Data collection procedures: SLS kinematics

Participants performed three separate SLSs on each leg with the starting leg determined by coin toss. For each SLS, participants were instructed to begin by standing on one leg with the opposite knee flexed to approximately 90 degrees, arms folded across their chest, and looking ahead. Participants were then asked to squat down on the weight-bearing limb in a slow controlled manner as far as possible without losing balance, before returning to the starting position. All participants were allowed a maximum of three practice attempts. In accordance with previous investigations [10,11,15], we chose not to standardize squat depth in keeping with an approach that most closely resembles clinical practice.

For each SLS, both two-dimensional video and three-dimensional motion analysis data were collected. Video data were collected with a high-definition digital video camera (Sony DCR-SX40E, Sydney, Australia) which was subsequently viewed at a later date for rating by both experienced physiotherapists and physiotherapy students. The video camera was positioned directly in front of the participant at a distance of three metres and a height of one metre. Each video recording was transferred to a personal computer and cropped (i.e. temporally) to ensure a consistent start and finish point for viewing each performance and avoid pre- and post-squat movements influencing ratings. The vertical position of the frame of reference excluded the head of each participant, but allowed unobstructed observation of the lower limbs, pelvis and trunk.

**Figure 1:**
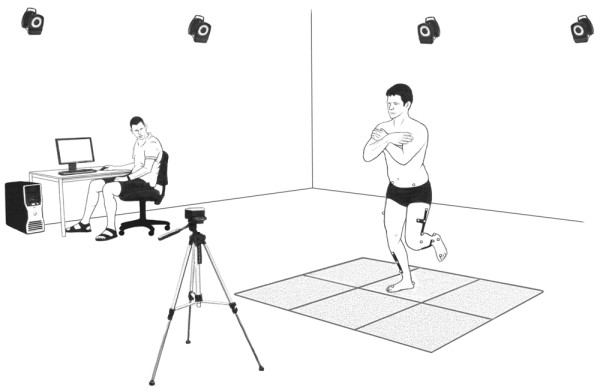
Marker placement and laboratory set up for single-leg squat motion capture and video recording.

Three-dimensional kinematic data were collected using a ten-camera VICON motion analysis system (MX13 Cameras, Oxford Metrics, Oxford, UK) operating at 200 Hz. Retro-reflective markers were attached to specific anatomical landmarks including the second metatarsal head, medial and lateral malleoli, calcaneus, medial and lateral femoral condyles, and right and left anterior superior and posterior superior iliac spines. In addition to individual markers, clusters of four markers were securely attached to the shank and thigh segments (Figure 1). Prior to kinematic data collection, a series of subject calibration trials were performed to determine anatomical landmarks, define lower limb joint coordinate systems and establish neutral joint positions. The calibration trials involved participants standing in anatomical position as well as undertaking a functional hip and knee joint movement task [18,19]. The functional movement task enabled the identification of hip joint centres by fitting a sphere to motion of the thigh markers and identification of knee joint flexion/extension axes using a mean helical axis method [18,20].

Raw three-dimensional coordinate data were filtered using a zero-lag fourth-order low-pass Butterworth filter, with a cut-off frequency of 8 Hz. Filtered marker trajectories were subsequently used to compute three-dimensional segment (pelvis) and joint (hip and knee) kinematics using BodyBuilder modelling software, version 3.6 (Vicon; Oxford Metrics). The convention used to describe kinematics was in accordance with the recommendations of the International Society of Biomechanics [21-23]. Segment and joint angles were calculated using the Euler angle method in a flexion/extension, abduction/adduction, and internal/external rotation sequence.

### Data collection procedures: physiotherapist ratings

Of the three SLSs performed on each leg, we chose to analyse the second squat of both the right and left leg for each participant, providing 44 SLSs in total. In a clinical setting, the SLS is typically rated on the performance of an individual squat rather than the average of multiple squats, providing support for this method of analysis. Following the identification of 44 SLSs for analysis, experienced musculoskeletal physiotherapists and physiotherapy students viewed the SLSs in random order on a personal computer. Raters were instructed to view each video twice at normal speed with no exceptions (i.e. no pausing or rewinding) and rate each performance. A 10-point ordinal scale was employed to emulate the clinical setting, with a score of 1 representing ‘very poor’ and a score of 10 representing ‘very good’. Ten ordinal points were preferred over minimal scales (e.g. good/fair/poor) to avoid ambiguities in the classification of performance quality. Similar scales have previously been used to classify gait quality from video footage [24]. To avoid ‘coaching’ or contamination of student and therapist perceptions, raters were not given guidelines on which to base their ratings. Rather, they were asked to simply rate the quality of the movement observed. Raters were further instructed not to discuss their own ratings with any other raters or participants.

In order to examine inter- and intra-rater reliability, all raters were invited to view the videos again two weeks later following the same instructions. Again, videos were viewed in random order.

### Data analysis

Data analyses were restricted to the combined up and down phase of each squat. The start of each squat was defined as the point where knee flexion angle changed by more than two degrees, while the end was defined as the point where minimal knee extension occurred following the up phase. The dependent variables used for statistical analyses were extracted from each trial using custom-designed software in Matlab 7.8.0 (The MathWorks, Natick, MA).

### Statistical analysis

Statistical analyses were performed using the Statistical Package for the Social Sciences (SPSS) version 20.0 for Windows (IBM, Chicago, IL, USA). Means and standard deviations were calculated for subject characteristics. Stepwise multiple linear regression analysis was used to determine joint kinematic predictors of SLS performance scores for experienced physiotherapists and physiotherapy students. Inter- and intra-rater reliability was determined using a two-way mixed model to generate intra-class correlation coefficients (ICC_3,1_) of consistency. Interpretation of ICC_3,1_ values was made according to the scale described by Rosner [25] (i.e. poor reliability 0–0.40; fair to good reliability 0.40-0.75; and excellent reliability 0.75-1.00). T-tests were used to compare scores from initial ratings to those from repeat ratings. Statistical significance was observed at *p* ≤ 0.05.

## Results

### Participant characteristics

A total of 22 healthy young adults (13 men and 9 women; 23.8 ± 3.1 years of age) consented to participate in the trial. Participants were 1.73 ± 0.07 m tall with a body mass of 69.4 ± 12.5 kg and body mass index of 22.9 ± 2.8 kg.m^-2^. Eight experienced post-graduate trained musculoskeletal physiotherapists and eight physiotherapy students in their penultimate year of study volunteered to rate the SLSs identified for analysis. Physiotherapists had 17.6 ± 7.7 years of clinical experience (range: 11 – 35 years).

### Physiotherapist ratings

Mean physiotherapist rating of SLS quality was 6.4 ± 1.3 on the first occasion and 6.4 ± 1.3 on the second occasion, while mean student rating of SLS quality was 6.0 ±1.6 on the first occasion and 6.0 ± 1.7 on the second occasion. Neither physiotherapist nor student ratings differed significantly between the two occasions.

### Kinematic predictors of SLS performance

Sex-specific analysis of joint kinematic data revealed no significant differences with the exception of peak knee flexion (Table 1). Thus, subsequent analyses were conducted on pooled data for the whole cohort. Average peak hip and knee joint kinematic values for all participants are presented in Table 2. Multiple regression analysis identified several kinematic predictors of SLS performance. Peak knee flexion explained 33% of the variance in physiotherapist ratings of SLS performance. The prediction was strengthened by 21% by adding peak hip adduction to the model, and a further 10% by the addition of knee medio-lateral displacement (Table 3). Peak knee flexion explained 36% of the variance in student ratings of SLS performance. The prediction was strengthened by a further 11% by adding knee medio-lateral displacement to the model (Table 4).

**Table 1:** Hip and knee joint kinematics (mean ±SD) for male and female participants

**Kinematic Variable**	** Men**	** Women**	**p-value**
Peak hip flexion (deg)	86.5 ± 10.6	76.2 ± 18.0	0.11
Peak hip adduction (deg)	15.5 ± 5.0	20.8 ± 7.1	0.06
Peak hip abduction (deg)	0.7 ± 4.0	2.4 ± 7.2	0.50
Peak hip external rotation (deg)	^−^15.1 ± 3.5	^−^15.7 ± 6.1	0.77
Peak hip internal rotation (deg)	^−^5.5 ± 3.2	^−^1.2 ± 7.4	0.08
Peak knee flexion (deg)	86.2 ± 13.0	71.5 ± 7.3	0.01
Knee medio-lateral displacement (mm)	44.8 ± 13.9	52.2 ± 22.7	0.35

**Table 2:** Hip and knee joint kinematics averaged across all SLS performances (n = 44)

**Kinematic Variable**	**Mean ± SD**	**Range (min** – **max)**
Peak hip flexion (deg)	83.4 ± 14.3	34.4 – 102.7
Peak hip adduction (deg)	17.7 ± 6.1	7.0 – 32.4
Peak hip abduction (deg)	1.7 ± 5.0	^−^9.2 – 14.9
Peak hip external rotation (deg)	^−^14.8 ± 4.8	^−^27.3 – ^−^6.1
Peak hip internal rotation (deg)	^−^3.3 ± 5.2	^−^11.9 – 15.3
Peak knee flexion (deg)	79.9 ± 12.3	57.3 – 110.9
Knee medio-lateral displacement (mm)	46.0 ± 16.1	15.1 – 89.8

**Table 3:** Regression models for kinematic predictors of SLS quality rated by experienced musculoskeletal physiotherapists

**Model**	**β-coefficient (SEM)**	**t**	**R**^**2**^	**p-value**
1 *constant*	^−^0.79 (1.59)	^−^0.50		
Peak knee flexion	0.09 (0.02)	4.59	0.334	0.01
2 *constant*	2.99 (1.60)	1.87		
Peak knee flexion	0.08 (0.02)	4.47		
Peak hip adduction	^−^0.15 (0.03)	^−^4.30	0.540	0.01
3 *constant*	5.08 (1.56)	3.25		
Peak knee flexion	0.07 (0.02)	4.37		
Peak hip adduction	^−^0.13 (0.03)	^−^0.40		
Knee medio-lateral displacement	^−^0.04 (0.01)	^−^0.33	0.639	0.01

**Table 4:** Regression models for kinematic predictors of SLS quality rated by physiotherapy students

**Model**	**β-coefficient (SEM)**	**t**	**R**^**2**^	**p-value**
1 *constant*	0.31 (1.19)	0.26			
Peak knee flexion	0.07 (0.02)	4.89	0.363	0.01	
2 *constant*	3.20 (1.17)	2.73			
Peak knee flexion	0.06 (0.01)	4.85			
Knee medio-lateral displacement	^−^0.04 (0.01)	^−^4.50	0.574	0.01	

### Reliability of ratings

Inter-rater reliability was good for physiotherapists (ICC_3,1_ = 0.71) and students (ICC_3,1_ = 0.60), while intra-rater reliability was excellent for physiotherapists (ICC_3,1_ = 0.81; range 0.66-0.87) and good for students (ICC_3,1_ = 0.71; range 0.50-0.87).

## Discussion

In establishing the utility of the SLS test in clinical practice, our goals were to (i) determine the lower limb joint kinematic variables that best predicted therapist ratings of SLS performance, (ii) evaluate whether kinematic predictors of SLS performance were influenced by rater experience, and (iii) assess the reliability of the SLS assessment for novice and experienced physiotherapists. We found that peak knee flexion angle was the strongest predictor of SLS performance and that knee joint medio-lateral displacement enhanced the multivariate model prediction of SLS performance for both experienced and student raters. The model prediction was further enhanced by the inclusion of peak hip adduction angle for experienced physiotherapists, indicating that they took into account both hip and knee movement when determining SLS performance. As a result, experienced physiotherapists demonstrated higher inter- and intra- rater reliability than their less experienced counterparts.

Around 70% of all ACL injuries are incurred through a non-contact mechanism [26]. Accumulating evidence suggests that undesirable movement patterns, such as excessive knee valgus, underpin the mechanism and heighten the risk of ACL injury [1,27]. The SLS test provides an attractive and clinically-efficient means of identifying undesirable movement patterns during screening and rehabilitation. However, before clinicians could apply and interpret the SLS test results with confidence, there was a need to establish the validity of clinical judgement related to the test. Our findings are important as they have established the convergent validity and reliability of the SLS test for both novice and experienced physiotherapists.

Peak knee flexion was the strongest predictor of SLS performance. Unlike others [7,9,13,14], we purposely chose not to standardize the depth of each SLS in an endeavour to reflect a typical clinical scenario. Despite all observations being made from directly in front of each subject, our results highlight the importance placed on adequate knee flexion by clinicians in making their assessment. In fact, knee flexion during the SLS is found to differ between men and women [11], which is supported by our sex-specific kinematic analysis, albeit in a relatively small sample. It appears necessary then that ratings are sensitive to this kinematic parameter. For studies investigating biomechanical differences (e.g. between sexes), however, we recommend standardizing knee flexion during the SLS.

In agreement with our hypothesis both physiotherapists’ and students’ scores were predicted by knee medio-lateral displacement. Not surprisingly, differences in knee motion in the frontal plane have been identified in different patient populations [28,29]. Indeed, frontal plane angular deviation of the thigh during the SLS is greater for patients with patellofemoral pain syndrome than for normal, healthy individuals [28]. Furthermore, ‘valgus collapse’ appears to be a common injury mechanism for non-contact ACL injury [30,31] and such observations are corroborated by laboratory-based studies of knee joint kinematics in ACL-injured patients [29]. Our findings demonstrate that physiotherapist and student assessment is capable of detecting medio-lateral position changes at the knee.

In partial agreement with our hypothesis, peak hip adduction was a significant predictor of SLS performance, however only for physiotherapists. In fact, predictors for students were only apparent at the knee. This distinction may be clinically important as significant differences in hip adduction have been reported for patient groups [2]. For example, Willson and Davis [2] report greater peak hip adduction for women with patellofemoral pain syndrome than for normal, healthy women. Interestingly, individuals who adopt a greater ‘dynamic knee valgus’ position during the SLS test, are more likely to exhibit greater hip adduction angles during dynamic activities such as running and jumping [2]. Such patterns of movement have been suggested to place those individuals at greater risk of musculoskeletal injury. Consequently, the ability to detect excessive hip adduction angle during the SLS test may be an important clinical screening skill.

We found that both physiotherapists and physiotherapy students were capable of reliable SLS assessment, although clinical experience enhanced both inter- and intra-rater reliability. The fact that physiotherapy students also demonstrated good within- and between-rater reliability in their assessments supports the robustness of the test itself. This finding is in support of Crossley and colleagues [10] who demonstrated similarity in ratings between experts and novices for the SLS test. Our results suggest that clinicians of all levels of experience can use the SLS test with confidence to identify undesirable movement patterns, at least in generally healthy individuals.

Although our design and testing protocol were intended to closely reflect routine clinical practice, several limitations warrant acknowledgement. Firstly, our sample included only healthy, young individuals comprising a mix of sexes. Exclusion of participants with lower limb pathology may be considered a limitation, though that significant findings were forthcoming highlights the sensitivity of the assessment. As we were primarily interested in the assessment of the SLS rather than a characterisation of the kinematics, reporting sex-specific data was not considered necessary. Secondly, the requirement for multiple raters and repeat measures necessitated the use of videos for rating, which might reduce the authenticity of the clinician-patient interaction. Finally, a relatively short period of time (i.e. two weeks) was used between the first and second ratings; however, our intention was to emulate a reasonable patient follow-up timeframe for maximum clinical applicability.

## Conclusion

In conclusion, we found that both physiotherapists and physiotherapy students were capable of reliably rating SLS performance in a cohort of healthy young adults. Further, we found that physiotherapist ratings were more strongly related to three-dimensional kinematics at both the hip and knee joint, as opposed to physiotherapy student ratings which were solely related to knee joint kinematics. In order to better understand the utility of the SLS test in clinical practice (and athletic screening), future work will investigate gender differences and the contribution of lower limb muscle activity in patient populations.

## Competing interest

Authors declare that there are no competing interests.

## Authors’ contributions

BW and SH contributed to the design of the study. BW, SH, and CC collected the data. SH and CC performed the data management. BW and SH performed the data analysis and were in charge of data interpretation. BW wrote the draft manuscript. All authors participated in data interpretation and contributed to manuscript revisions. All authors read and approved the final version.

## Pre-publication history

The pre-publication history for this paper can be accessed here:

http://www.biomedcentral.com/1471-2474/13/207/prepub
